# Unveiling a Novel MT-TS1 m.7479G>A in Mitochondrial Diabetes: The Critical Role of mtDNA Sequencing in Atypical Cases

**DOI:** 10.1210/jcemcr/luaf341

**Published:** 2026-01-28

**Authors:** Eleanor Danek, Felicity Pyrlis, Aleena Shujaat Ali, Elif I Ekinci

**Affiliations:** Department of Endocrinology and Centre for Research in Education in Diabetes and Obesity (CREDO), Austin Health, Heidelberg, VIC 3084, Australia; Department of Endocrinology and Centre for Research in Education in Diabetes and Obesity (CREDO), Austin Health, Heidelberg, VIC 3084, Australia; Department of Endocrinology and Centre for Research in Education in Diabetes and Obesity (CREDO), Austin Health, Heidelberg, VIC 3084, Australia; Department of Medicine, University of Melbourne, Fitzroy, VIC 3065, Australia; Australian Centre for Accelerating Diabetes Innovations, School of Medicine, University of Melbourne, Parkville, VIC 3052, Australia; Department of Endocrinology and Centre for Research in Education in Diabetes and Obesity (CREDO), Austin Health, Heidelberg, VIC 3084, Australia; Department of Medicine, University of Melbourne, Fitzroy, VIC 3065, Australia; Australian Centre for Accelerating Diabetes Innovations, School of Medicine, University of Melbourne, Parkville, VIC 3052, Australia

**Keywords:** mitochondrial diabetes, *MT-TS1*, m.7479G>A, genetic testing, maternal inheritance, atypical diabetes

## Abstract

Mitochondrial diabetes is a rare form of diabetes mellitus caused by mitochondrial DNA (mtDNA) mutations, often presenting with atypical features and maternal inheritance. We report a 71-year-old white female presenting with diabetes diagnosed at age 50, managed with oral therapy, who exhibited significant weight loss and a strong maternal family history of diabetes. Glutamic acid decarboxylase and insulinoma-associated-2 antibodies were negative with normal C-peptide, and genetic testing revealed a heteroplasmic *MT-TS1* m.7479G>A variant (13.90%). Glycemic management was achieved with metformin and gliclazide, and at 21 years post diagnosis, the patient maintained stable glycemic control with a glycated hemoglobin A_1c_ of 6.5% (SI: 48 mmol/mol) (reference range, 4.0%-6.0% [SI 20-42 mmol/mol]) without insulin. The *MT-TS1* m.7479G>A variant is implicated as a pathogenic cause of mitochondrial diabetes, highlighting the importance of mtDNA sequencing in atypical cases with maternal inheritance, the potential for milder phenotypes with low-heteroplasmy variants, and the critical role of genetic counseling.

## Introduction

Mitochondrial diabetes is a rare monogenic form of diabetes, accounting for less than 1% of diabetes cases, resulting from mitochondrial DNA (mtDNA) pathogenic variants that impair β-cell function and insulin secretion [1]. Unlike nuclear DNA mutations seen in maturity-onset diabetes of the young (MODY), pathogenic variants in mtDNA exhibit maternal inheritance due to exclusive maternal transmission of mitochondria [2]. These pathogenic variants disrupt mitochondrial oxidative phosphorylation and reduce adenosine triphosphate (ATP) production, which is critical for glucose-stimulated insulin release [1]. The most common variant, m.3243A>G in the *MT-TL1* gene, is associated with maternally inherited diabetes and deafness, characterized by diabetes onset in young adulthood, sensorineural hearing loss, and occasionally neuromuscular symptoms [3].

**Figure 1.**
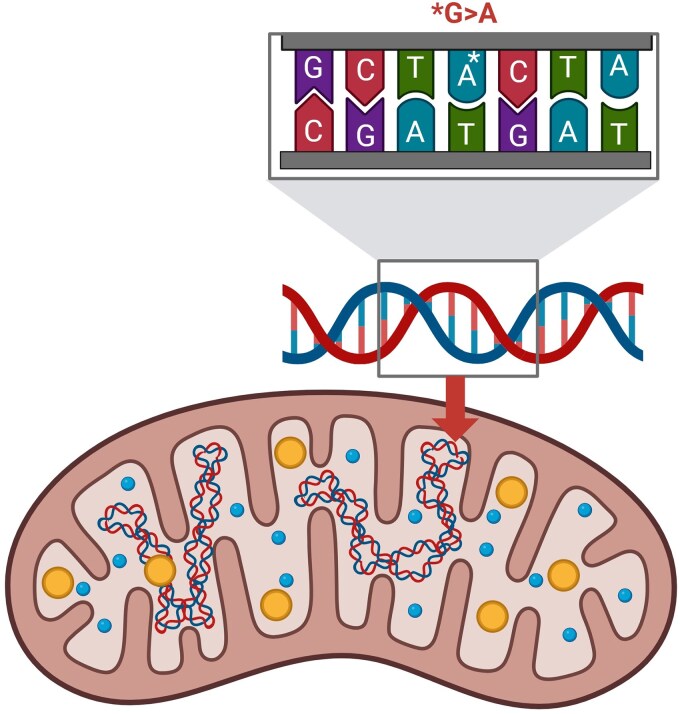
Diagram of a mitochondrion highlighting the *MT-TS1* m.7479G>A mutation. This novel variant involves a guanine-to-adenine substitution at position 7479 in the mitochondrial transfer RNA (tRNA) serine 1 (*MT-TS1*) gene, likely disrupting tRNA function and contributing to mitochondrial diabetes.

The *MT-TS1* gene encodes a mitochondrial transfer RNA (tRNA) for serine, which is crucial for protein synthesis within the mitochondria. Specifically, the *MT-TS1* gene facilitates the incorporation of serine into proteins encoded by mtDNA during translation, a process vital for the assembly of functional mitochondrial respiratory chain complexes [4]. These complexes are essential for oxidative phosphorylation, the metabolic pathway that produces ATP to support cellular functions, including insulin secretion in β cells [1]. Pathogenic variants in *MT-TS1* like m.7479G>A may disrupt tRNA function, potentially impairing mitochondrial protein synthesis and reducing ATP production, which could contribute to β-cell dysfunction and diabetes [4] (Fig. 1).

The HmtVar database serves as a critical reference for researchers and clinicians to assess the clinical significance of mtDNA variants by comparing their prevalence in healthy vs diseased populations and evaluating their predicted effect on mitochondrial function [4]. The absence of the m.7479G>A variant in HmtVar suggests it is either extremely rare or not well characterized, limiting insights into its prevalence or pathogenic potential. Additionally, in silico tools in the MitoTIP database, which use computational algorithms to predict the potential pathogenicity of mitochondrial tRNA variants based on factors such as the structural effect of tRNA, provide uncertain evidence of pathogenicity for this variant [5]. Table 1 summarizes the existing case reports of known pathogenic variants in the *MT-TS1* gene [6-12]. To date, the m.7479G>A variant has not been reported in the literature in patients with mitochondrial disease, underscoring the need for further research to clarify its clinical significance [4, 5].

**Table 1.** Known pathogenic mutations in *MT-TS1* from the HmtVar database [4]

Variant (HGVS)	HmtVar accession	Clinical significance	Associated phenotype(s)	Heteroplasmy levels	Functional effect	Reference(s)
m.7445A>G	VCV000018984	Pathogenic	Non-syndromic sensorineural deafness, palmoplantar keratoderma with deafness	Not specified	Disrupts tRNA production, reducing mitochondrial protein synthesis	[6, 7]
m.7472Cins	VCV000018986	Pathogenic	MERRF, progressive myoclonic epilepsy	Not specified	Alters tRNA structure, reducing translation efficiency	[8]
m.7512T>C	VCV000018985	Pathogenic	MERRF/MELAS overlap syndrome, MERRF-like syndrome	93% in muscle, 76%-87% in blood	Disrupts tRNA acceptor stem	[9, 10]
m.7497G>A	VCV000018987	Pathogenic	Mitochondrial myopathy	Not specified	Causes tRNA depletion due to impaired maturation	[10, 11]
m.7505T>C	VCV000018988	Pathogenic	Nonsyndromic hearing loss	Homoplasmic	Reduces tRNA levels to ∼35% of controls	[12]

Abbreviations: MELAS, mitochondrial encephalomyopathy, lactic acidosis, and stroke-like episodes; MERRF, myoclonic epilepsy with ragged red fibers; tRNA, transfer RNA.

Mitochondrial diabetes often presents with atypical features, including significant weight loss, absence of autoantibodies, and preserved C-peptide, challenging conventional diabetes classification [1, 3]. Identifying such variants is crucial for accurate diagnosis, guiding treatment and offering genetic counseling. We present a case of mitochondrial diabetes in a 71-year-old woman with a previously undescribed variant in *MT-TS1* m.7479G>A, proposing its potential pathogenic role. This case highlights the importance of genetic testing in atypical diabetes presentations to inform diagnosis, tailor management, and advance understanding of rare mtDNA variants.

## Case Presentation

**Figure 2.**
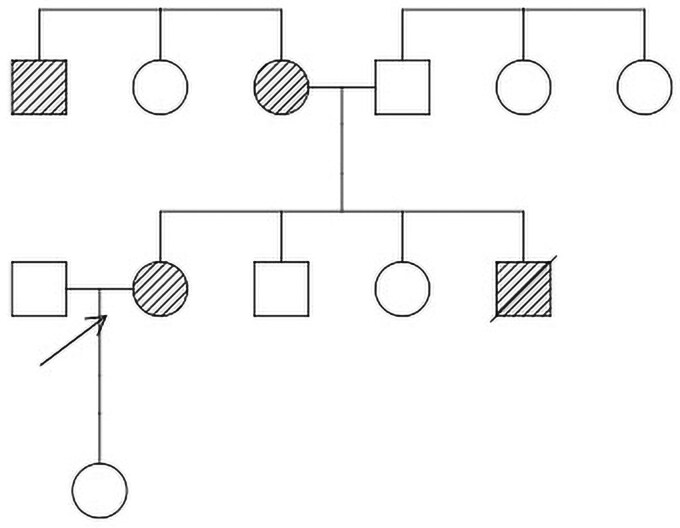
Pedigree chart showing maternal inheritance of diabetes. The proband, our patient, is marked with an arrow. Those known to be affected with diabetes are shaded. Confirmed mitochondrial diabetes is the proband, and likely mitochondrial diabetes in her brother, mother, and maternal uncle.

A 71-year-old White woman was diagnosed with diabetes at age 50 years by her general practitioner (GP), after presenting with fatigue, in the absence of more specific symptoms of hyperglycemia such as polyuria or polydipsia. Her mother was diagnosed with presumed type 1 diabetes at age 36 and was commenced on insulin, as were her maternal uncle (diagnosed in his 30s) and brother (diagnosed at age 55, died at age 60 of myocardial infarction) (Fig. 2). At diagnosis, the patient’s weight was 70 kg (BMI 26). Comorbidities included hypercholesterolemia, hypertension, drug-induced hepatitis from statin therapy (whereby progressive derangement in liver function tests occurred in the months following statin initiation and fully resolved within 3 months of cessation), Hashimoto hypothyroidism, and an intraductal papillary mucinous neoplasm of the pancreas (IPMN), likely unrelated to her mitochondrial diabetes. She has no history of hearing loss. Over 21 years, she experienced significant weight loss to 46 kg (BMI 17.10), without diabetes resolution. She had no history of ketoacidosis or hypoglycemic episodes. Her diabetes was managed with oral therapy (metformin 1000 mg daily, sitagliptin 100 mg daily) since diagnosis, and she had never required insulin prior to referral to an endocrinologist.

## Diagnostic Assessment

Initial laboratory evaluation at diagnosis revealed a glycated hemoglobin A_1c_ (HbA_1c_) of 9.0% (75 mmol/mol), and a fasting glucose of 180 mg/dL (SI: 10.0 mmol/L) (reference range, 70-99 mg/dL [SI: 3.9-5.5 mmol/L]). Twenty-one years later at age 71, in the setting of suboptimal glycemic control (HbA_1c_ of 7.4% [SI: 57 mmol/mol]) and referral to an endocrinologist, further testing was undertaken to clarify diabetes etiology. Investigations revealed negative glutamic acid decarboxylase (GAD) and insulinoma-associated-2 (IA2) antibodies, and a normal C-peptide level of 0.8 nmol/L (reference range, 0.4-1.5 nmol/L), suggesting preserved β-cell function. Zinc transporter 8 (ZnT8), islet cell (ICA), and insulin (IAA) autoantibodies were not performed due to local laboratory protocols at the time, which prioritized GAD and IA2 as first-line markers for adult-onset autoimmune diabetes, consistent with regional guidelines and resource availability. Given the maternal family history and atypical presentation, genetic testing for monogenic diabetes was performed via next-generation sequencing of mtDNA for the genes included in the Blueprint Genetics MODY Panel (version 6, October 30, 2021). A heteroplasmic *MT-TS1* m.7479G>A variant (13.90%) was identified, absent from major databases (MitoTIP and HmtVar) [3, 4] and previously undescribed in the literature. Liver function tests were normal despite prior statin-induced hepatitis, and thyroid function was stable on levothyroxine.

## Treatment

The patient's diabetes was managed with metformin (500 mg twice daily), and sitagliptin was ceased in the setting of IPMN (due to a theoretical risk of ductal cell proliferation) after referral to an endocrinologist. In the setting of suboptimal glycemic control on metformin monotherapy (HbA_1c_ of 7.4% [SI: 57 mmol/mol]) and significant weight loss down to a BMI of 17.10, gliclazide (modified release) was introduced and titrated to 60 mg daily. Despite existing literature cautioning the use of metformin in mitochondrial diabetes due to risk of lactic acidosis, it was continued after diagnosis given no adverse effects over 21 years. Coenzyme Q10 (CoQ10) supplementation, which has been proposed to support electron transport chain function in mitochondrial disorders, was not initiated in this patient because currently available evidence, including a Cochrane systematic review, does not demonstrate clear clinical benefit to justify routine use [13]. Hypertension was managed with perindopril, and hypercholesterolemia was managed with ezetimibe post statin intolerance. She maintained euthyroidism on levothyroxine. Genetic counseling was offered to relatives, given the maternal inheritance pattern, and her daughter has elected to pursue testing.

## Outcome and Follow-up

At 21 years post diagnosis and 3 months after the addition of gliclazide, the patient achieved glycemic control (HbA_1c_ of 6.5% [SI: 48 mmol/mol]). In addition, a healthy weight gain of 5 kg over 6 months was observed after initiation of gliclazide, with improvement in BMI from 17.10 to 19.0. No complications (retinopathy, neuropathy, nephropathy), episodes of hypoglycemia or lactic acidosis occurred. Routine monitoring included HbA_1c_ and renal function measurement every 3 to 6 months, and annual ophthalmology review. Her pancreatic IPMN remained stable on imaging. Follow-up continues with her endocrinologist and GP, with no insulin requirement.

## Discussion

This case report describes a 71-year-old woman with mitochondrial diabetes associated with a previously undescribed variant in *MT-TS1* m.7479G>A, characterized by late-onset diabetes, significant weight loss, maternal inheritance, and sustained glycemic control without insulin. The presentation diverges from classic mitochondrial diabetes, such as that caused by the m.3243A>G variant in *MT-TL1*, which typically includes sensorineural hearing loss and earlier onset (20-40 years) of diabetes [1, 3]. The absence of hearing loss in this patient and the novel nature of the *MT-TS1* m.7479G>A variant suggests a distinct clinical profile, potentially influenced by its low heteroplasmy level (13.90%). The *MT-TS1* gene encodes a mitochondrial tRNA for serine (tRNASer(UCN)) which is essential for incorporating serine into proteins during mitochondrial translation [14]. The novel m.7479G>A variant, located in *MT-TS1*, may impair tRNASer(UCN) function, potentially disrupting its secondary or tertiary structure, aminoacylation, or posttranscriptional maturation, as observed with other *MT-TS1* variants like m.7445A>G [6, 7] and m.7497G>A [11]. Such disruptions can reduce the synthesis of OXPHOS complexes, leading to decreased ATP production in pancreatic β cells, which are critical for glucose sensing and insulin secretion. The low heteroplasmy level (13.90%) of m.7479G>A (noting that blood tissue, as used in this patient's case, often has lower heteroplasmy levels than salivary tissue) may contribute to the milder, late-onset diabetes phenotype without sensorineural hearing loss, in contrast to high-heteroplasmy variants like m.3243A>G in *MT-TL1*, which often cause more severe symptoms [3, 15]. The *MT-TS1* m.7479G>A variant's pathogenicity remains unconfirmed due to limited functional studies and its absence from major genetic databases (MitoTIP, HmtVar) [4, 5]. However, pathogenicity is highly likely given the patient's presentation, which is consistent with mitochondrial diabetes, including maternal inheritance, significant weight loss, late-onset diabetes without insulin dependence, and an atypical diabetes phenotype, as well as the *MT-TS1* gene's critical role in mitochondrial tRNA function and oxidative phosphorylation.

This case contrasts sharply with MODY, which involves autosomal dominant nuclear gene mutations (eg, *HNF1A*, *GCK*) [16]. The patient's normal C-peptide level after 21 years effectively excluded type 1 diabetes and phenotypic variability inconsistent with type 2 diabetes supported a monogenic etiology, prompting genetic testing. The identification of the *MT-TS1* m.7479G>A variant underscores the diagnostic challenge of atypical diabetes presentations, particularly in older patients, in whom type 2 diabetes is often assumed. With genetic testing increasingly accessible in the modern clinical setting, such cases highlight the need to consider mitochondrial diabetes in patients with maternal family histories, even without classic features like hearing loss. The significant weight loss (from BMI 26 to 17.10) observed here is likely due to impaired mitochondrial energy metabolism, as defective oxidative phosphorylation reduces ATP availability, leading to increased lipolysis and a catabolic state [17]. This is further supported by the patient's low heteroplasmy, which may preferentially affect energy-demanding tissues like adipose tissue and β cells, contributing to reduced adiposity [1]. Notably, after initiating gliclazide, the patient experienced welcomed weight gain (∼3-5 kg, pending confirmation with the patient), likely due to enhanced insulin secretion promoting an anabolic state, counteracting the catabolic effects of mitochondrial dysfunction [18].

A notable aspect of this case is the patient's long-term use of metformin, typically contraindicated in mitochondrial diabetes due to the risk of lactic acidosis [3, 19]. Metformin inhibits hepatic gluconeogenesis and may increase lactate production via inhibition of mitochondrial complex I; in the context of primary mitochondrial dysfunction, this can impair lactate clearance and elevate risk of type B lactic acidosis, particularly at high heteroplasmy or with renal impairment [19]. In our patient, the absence of lactic acidosis over 21 years suggests that metformin may be safe in select cases with low-heteroplasmy variants, preserved renal function, and close monitoring. This observation challenges current practice and warrants further investigation into personalized treatment strategies for mitochondrial diabetes.

The case has implications for clinical practice, particularly in Australia, where rural and remote populations may face barriers to genetic testing. Endocrinologists should maintain a high index of suspicion for mitochondrial diabetes in patients with atypical features, such as maternal inheritance or unexplained weight loss, and advocate for mtDNA sequencing when standard tests (eg, autoantibody panels, C-peptide) are inconclusive. Genetic counseling is critical, as mitochondrial variants carry a 100% transmission risk to offspring of affected females, though phenotypic expression varies due to heteroplasmy [2, 3]. Genetic counseling was offered to relatives, given the importance of family engagement in such cases.

Limitations of this report include the variant's uncertain pathogenicity, as functional studies (eg, cybrid cell models) are needed to confirm its effect on mitochondrial function. The lack of family testing to assess segregation of the variant and diabetes phenotype is another constraint, as is the absence of longitudinal data on heteroplasmy levels, which may fluctuate over time [1]. Furthermore, the incomplete autoantibody panel, with the absence of ZnT8, ICA, and IAA testing, should be noted. GAD is the most sensitive marker for latent autoimmune diabetes of adults in adults (>92%) [20, 21] of cases and its absence, combined with negative IA2 antibodies and long-term C-peptide preservation and identification of a pathogenic mtDNA variant, strongly argues against autoimmunity. Nonetheless, future cases should include a full panel where feasible to optimize the detection of type 1 diabetes mellitus. Future research should focus on characterizing rare mtDNA variants and establishing thresholds for metformin safety in mitochondrial diabetes. This case adds to the limited literature on *MT-TS1* variants and supports the growing role of genetic testing in precision endocrinology.

## Learning Points

- Mitochondrial diabetes should be considered in people presenting with atypical diabetes with maternal inheritance.
- Genetic testing for rare mtDNA variants is essential when standard diagnostic tests (eg, antibodies, C-peptide) are inconclusive.
- Low-heteroplasmy mitochondrial pathogenic variants may present with milder phenotypes, expanding the clinical spectrum of mitochondrial diabetes.
- Metformin may be cautiously continued in people with mitochondrial diabetes with low heteroplasmy.
- Genetic counseling is crucial to address the maternal transmission of mtDNA variants and support family screening.

## Contributors

E.D. prepared all clinical data, obtained patient consent, and drafted the manuscript; E.I.E. was involved in patient management; and E.D., E.I.E., F.P., and A.S. were involved in interpretation of clinical data, critical revision of the manuscript for important intellectual content, and final approval of the manuscript. All authors agreed to be accountable for all aspects of the work.

## Data Availability

Data sharing is not applicable to this article as no datasets were generated or analyzed during the current study.

## Abbreviations

ATP: adenosine triphosphate
BMI: body mass index
GAD: glutamic acid decarboxylase
GP: general practitioner
HbA_1c_: glycated hemoglobin A_1c_
IA2: insulinoma-associated-2 autoantibodies
IAA: insulin autoantibodies
ICA: islet cell autoantibodies
IPMN: intraductal papillary mucinous neoplasm of the pancreas
MELAS: mitochondrial encephalomyopathy, lactic acidosis, and stroke-like episodes
MERRF: myoclonic epilepsy with ragged red fibers
MODY: maturity-onset diabetes of the young
mtDNA: mitochondrial DNA
tRNA: transfer RNA
ZnT8: zinc transporter 8
